# Comparison of three-dimensional maxillary growth across spheno-occipital synchondrosis maturation stages

**DOI:** 10.1186/s12903-023-02774-w

**Published:** 2023-02-14

**Authors:** Waseem S. Al-Gumaei, Hu Long, Reem Al-Attab, Sadam A. Elayah, Maged S. Alhammadi, Ibtehal Almagrami, Remsh K. Al-Rokhami, Wenli Lai, Yan Zheng

**Affiliations:** 1 Department of Orthodontics and Dentofacial Orthopedics, School of Stomatology, Lanzhou University, Lanzhou, China; 2 State Key Laboratory of Oral Diseases and National Clinical Research Center for Oral Diseases and Department of Orthodontics, West China Hospital of Stomatology, Sichuan University, Chengdu, China; 3 Department of Dental Implant, School of Stomatology, Lanzhou University, Lanzhou, China; 4 State Key Laboratory of Oral Diseases and National Clinical Research Center for Oral Diseases and Department of Oral and Maxillofacial Surgery, West China Hospital of Stomatology, Sichuan University, Chengdu, China; 5 Orthodontics and Dentofacial Orthopedics, Department of Preventive Dental Sciences, College of Dentistry, Jazan University, Jazan, Saudi Arabia; 6 Department of Orthodontics, Faculty of Dentistry, First Affiliated Hospital of Zhengzhou University, Henan, China; 7 Department of Orthodontics, School of Stomatology, China Medical University, Shenyang, Liaoning China

**Keywords:** Maxillary growth, Spheno-occipital synchondrosis, Stages of fusion, CBCT, Three-dimensional

## Abstract

**Background:**

This study aimed to three-dimensionally compare the maxillary growth among the spheno-occipital synchondrosis (SOS) maturation stages in both genders.

**Methods:**

This is a cross-sectional study of a retrospective type in which cone-beam computed tomography (CBCT) images of 500 patients aged 6 to 25 years (226 males and 274 females) were analyzed. The SOS was evaluated using the four-stage scoring system; completely open, partially fused, semi-fused, or completely fused. The SOS scoring and three-dimensional cephalometric measurements were analyzed by Invivo 6.0.3 software. Descriptive and analytical statistics were performed and a *P*-value < 0.05 was considered statistically significant.

**Results:**

There was a statistically significant difference in maxillary measurements among SOS maturation stages in both genders (*P* < 0.05). The mean differences in the maxillary growth among the SOS maturation stages between SOS stages 2 and 3 were higher than those between stages 1and 2 and stages 3 and 4 for maxillary length and height in both genders. However, the mean difference in the maxillary width was higher between SOS stages 1 and 2 than those stages 2 and 3 and stages 3 and 4. On other hand, there may be lesser maxillary growth between SOS stages 3 and 4 for maxillary width, length (in males), and height. The growth curves showed high active growth of the maxilla as the SOS was still fusing (especially stage 2 and 3) than those of the fused (stage 4). Moreover, the acceleration of growth occurred earlier in females than males regarding chronological age but not for SOS maturation stages.

**Conclusions:**

The SOS maturation stages are valid and reliable maxillary skeletal maturation indicators for three-dimensional maxillary growth in both genders.

**Supplementary Information:**

The online version contains supplementary material available at 10.1186/s12903-023-02774-w.

## Introduction

The spheno-occipital synchondrosis (SOS) located in the midline between the sphenoid and occipital bones and considered the most important growth center in the cranial base because of its late ossification and contribution to post-natal cranial base growth [1, 2]. The cranial base is the template for facial development; therefore, it is directly related to the maxillary and mandibular growth and displacement. In individuals with craniofacial syndromes like Apert, Crouzon, Down, or Pfeiffer syndromes, the SOSs showed early ossification which correlated with a shorter cranial base and midface hypoplasia [3, 4].

The evaluation of craniofacial skeletal growth has critical importance in orthodontic, dentofacial orthopedic, orthognathic diagnosis, treatment planning, and evaluation of treatment result’s prognosis and stability [5, 6]. The main area of interest for the orthodontist is to know whether a patient has attained peak pubertal growth or passed that point. This, in turn, determines whether growth modification is still a viable treatment option [7, 8].

The most used craniofacial skeletal maturation indicators were hand-wrist (HW) and cervical vertebrae maturation (CVM) methods. However, each method has its inherited limitations [9, 10]. The hand-wrist method requires expert knowledge, expenditure of time by the operator, the method’s accuracy is not very high, and it exposes patients to an unnecessary dose of radiation [11]. The CVM method possesses poor reproducibility attributed to the level of training, clinician experience, and assessment methods [12–14]. Furthermore, the CVM method could not predict the amount of craniofacial growth in girls with Class II malocclusion [15, 16]. It is generally believed in the orthodontic community that there is still a need for a reliable skeletal maturity indicator that shows efficacy in detecting craniofacial growth and should not depend on only one skeletal indicator in clinical decisions [17–19]. Based on recent high-level evidence the CVMI and HW methods still not guarantee to provide a reliable tool for skeletal age assessment and it was recommended that further studies are warranted to confirm these findings or to validate another more effective tool and it was also suggested to use a combination of maturation signs along with developmental stages of cervical vertebrae in order to determine skeletal maturation until a quantitative and valid method is presented [11, 20].

The CBCT images provide accurate three-dimensional anatomical details and facilitate visualization of small osseous structures and high-resolution images compared to conventional radiographs [21]. Recently, the SOS method has been considered a valid and reliable indicator of skeletal age compared with the CVM, HW methods, and chronological age [10, 22–26]. Jabour studied mandibular growth and the SOS fusion stages [22], but no study in the available literature related it to 3D maxillary growth during the SOS fusion stages. Therefore, this study aimed to evaluate three-dimensional maxillary growth during SOS fusion stages in both genders, assessing the reliability of the SOS method as a skeletal indicator of 3D maxillary growth, calculating maxillary growth potential (mean differences), and constructing a basic maxillary growth curve.

## Materials and methods

### Sample selection

This cross-sectional study of a retrospective type was approved by the Ethics Committee of the School of Stomatology at Lanzhou University in a group of the Chinese population (No: LZUKQ-2019–042). The sample size primarily depended on previous studies [27, 28]. Because, either unilateral or bilateral maxillary constriction are commonly seen in daily orthodontic practice and the early intervention of this form of malocclusion in proper timing using either slow or rapid palatal expansion is always applicable and resulted in a great improvement and prevents further aggressive interventions in the adult age [29, 30], so maxillary width variable was selected for sample size calculation. The G* power 3.0.10 software (ver. 3.1.9.7; Heinrich-Heine-Universität Düsseldorf, Düsseldorf, Germany) was used to calculate the sample size; the a priori sample size estimation, performed at a 5% level of significance (*α* = 0.05), with a power of 99%, with mean values of width of the maxilla were 59.80 ± 3.31 mm in SOS stage I and 62.99 ± 2.93 mm in SOS stage II, effect sizes (*d* = 1.02), and a two-sided test comparing two independent samples. The calculation revealed that a minimum of 37 subjects were necessary per SOS stage group (four groups for each gender).

Data were randomly collected based on the pre-existing records between January 2016 and July 2021 according to a known patient age, sex, dental and medical history, and CBCT scan*.* The inclusion criteria were (1) age range from 6 to 25 years in which the upper and lower limits were determined following previous studies [31–33]; and (2) clear reporting of sex, dental and medical history. Exclusion criteria included (1) patients with reported cleft lip or cleft palate; (2) craniofacial syndromes; (3) head trauma and/or deformity; (4) gross asymmetry; (5) previous orthodontic or orthopedic treatment, or (6) inadequate diagnostic quality radiographs. The data of 572 subjects were collected, of which 72 were excluded; the remaining five hundred subjects, 274 females and 226 males were included in this study. Sample grouping was based on SOS scoring into four groups for each gender. The CBCTs were taken for evaluation of delayed eruption teeth, root resorption, survey whole dentition, third molar extraction, and diagnosis of nasomaxillary complex problem.

Informed consents were obtained from all subjects and their parents or legal guardians. Moreover, all methods were carried out in accordance with the principles of the declaration of Helsinki.

### Three-dimensional imaging

#### CBCT acquisition

CBCT images were acquired using I-CAT Imaging System (Imaging Sciences International Inc. Hatfield, USA). Each patient was scanned using a standard protocol that included a standardized head position, maximal intercuspation with the Frankfort horizontal plane parallel to the floor with a crossing laser guide. According to the imaging protocol, the patient was instructed not to swallow or move during the scanning process. The acquisition parameters used were: 16 × 13 cm field of view, 120 kV, 18.54 mAs, and 8.9 s exposure time. The selected voxel dimension was 0.3 mm, and the slice thickness was 2 mm.

#### SOS fusion staging

**Fig. 1:**
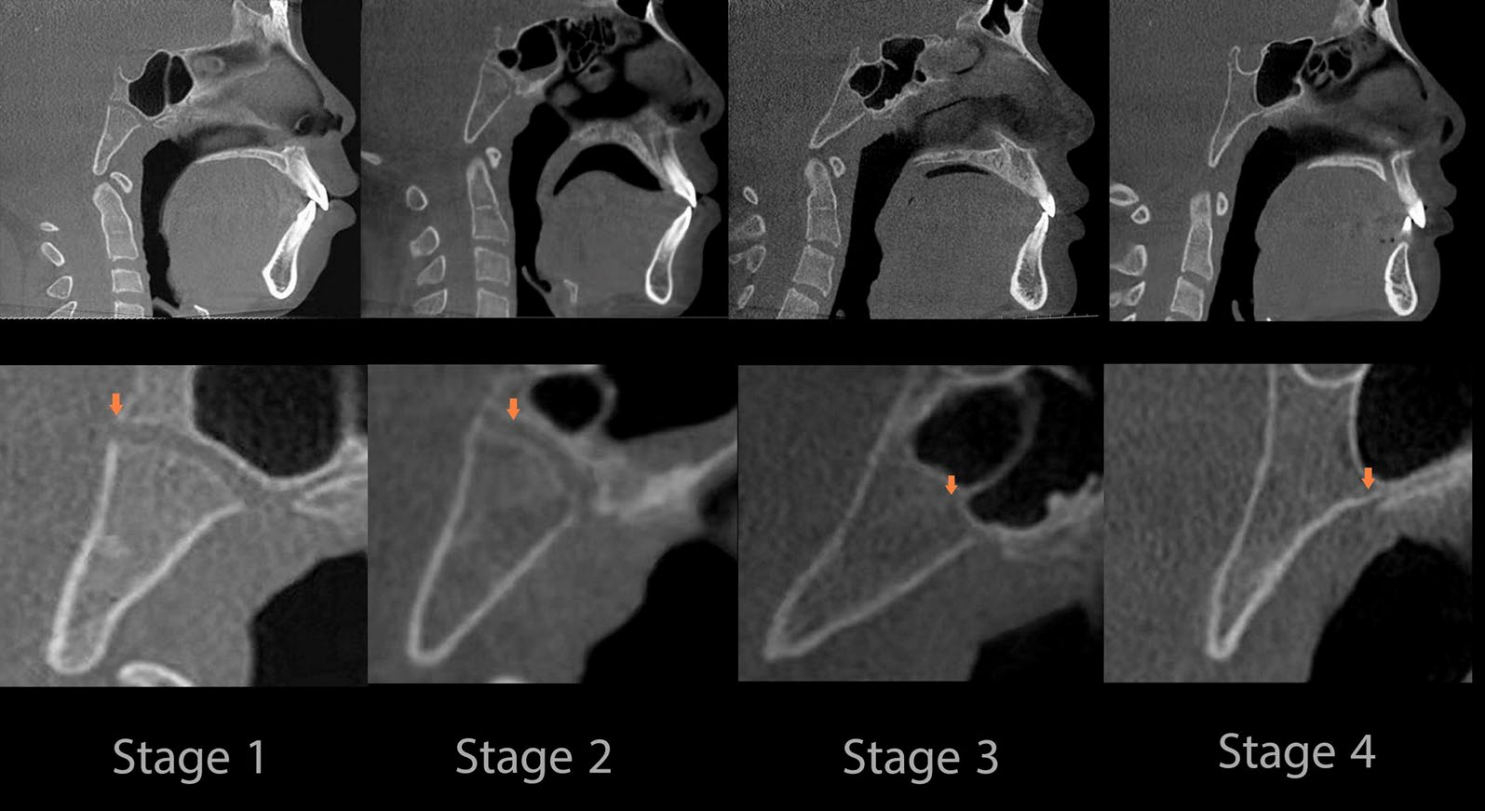
Stages of SOS fusion in the mid-sagittal plane (3D multi-planar reconstruction)

**Fig. 2:**
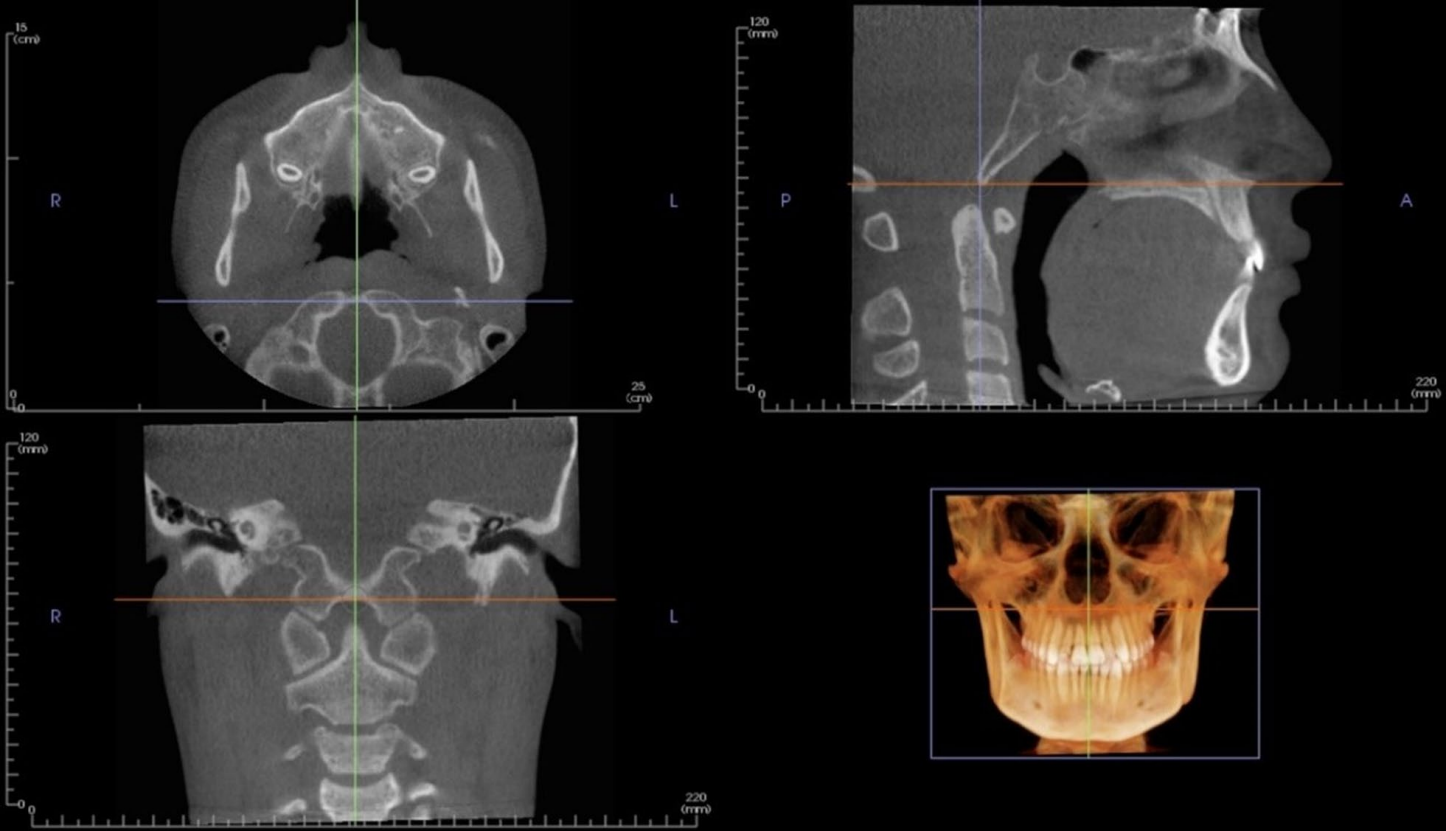
Mid-sagittal CBCT evaluation of spheno-occipital synchondrosis with the head in the proper orientation

Digital Imaging and Communications in Medicine (DICOM) files of the CBCT images were obtained and then imported into Invivo 6.0.3 software (Anatomage, San Jose, CA, USA). The spheno-occipital synchondrosis four-stage system of Franklin and Flavell [31] (Table 1 & Fig. 1**)** was followed. Lottering et al. [34] 6-stage SOS scoring system assumes the presence of fusion scar, which might persist for decades after fusion, has occurred [35]. Moreover, the four-stage scoring approach reduces assessment subjectivism, resulting in increased inter-observer agreement [31]. Moreover, it may be easier and needs less training by clinician and recommended by previous studies [24, 31, 32, 36, 37]. All 3D virtual models were oriented at a standardized position, then adjusted to the mid-sagittal plane (MSP) view (Fig. 2**)** [24, 31–33, 38, 39]. All CBCTs were assessed blindly with a coding system to mask the patient's demographic data and recorded in a separate data extraction sheet. Two well-trained observers, W.A. and R.A. independently scored the entire sample. Separated by a 1-month interval, both observers randomly selected 100 images and re-evaluated for intra-observer and inter-observer agreement of SOS staging. In the cases of disagreement, the axial view was used to assess the synchondrosis to reach a consensus as recommended by Okamoto et al. [40].

**Table 1:** Description of the fusion of the spheno-occipital synchondrosis scoring

Stage	Status	Description
1	Un-fused	Opened entirely with no sign of closure or presence of bone in the gap between the endocranial and ectocranial borders
2	Partial-fused	Fused endocranially but not more than half the length of the synchondrosis (Fusing endocranially, ≤ 50%)
3	Semi-fused	Fusing ectocranially with more than half the length of the synchondrosis but without fusing of the inferior (ectocranial) border. (Fusing ectocranially, > 50% and less than 100% fusion)
4	Complete fusion	Fused entirely with normal bone appearance throughout the synchondrosis, but a fusion scar may be existing

#### Three-dimensional measurements

**Fig. 3:**
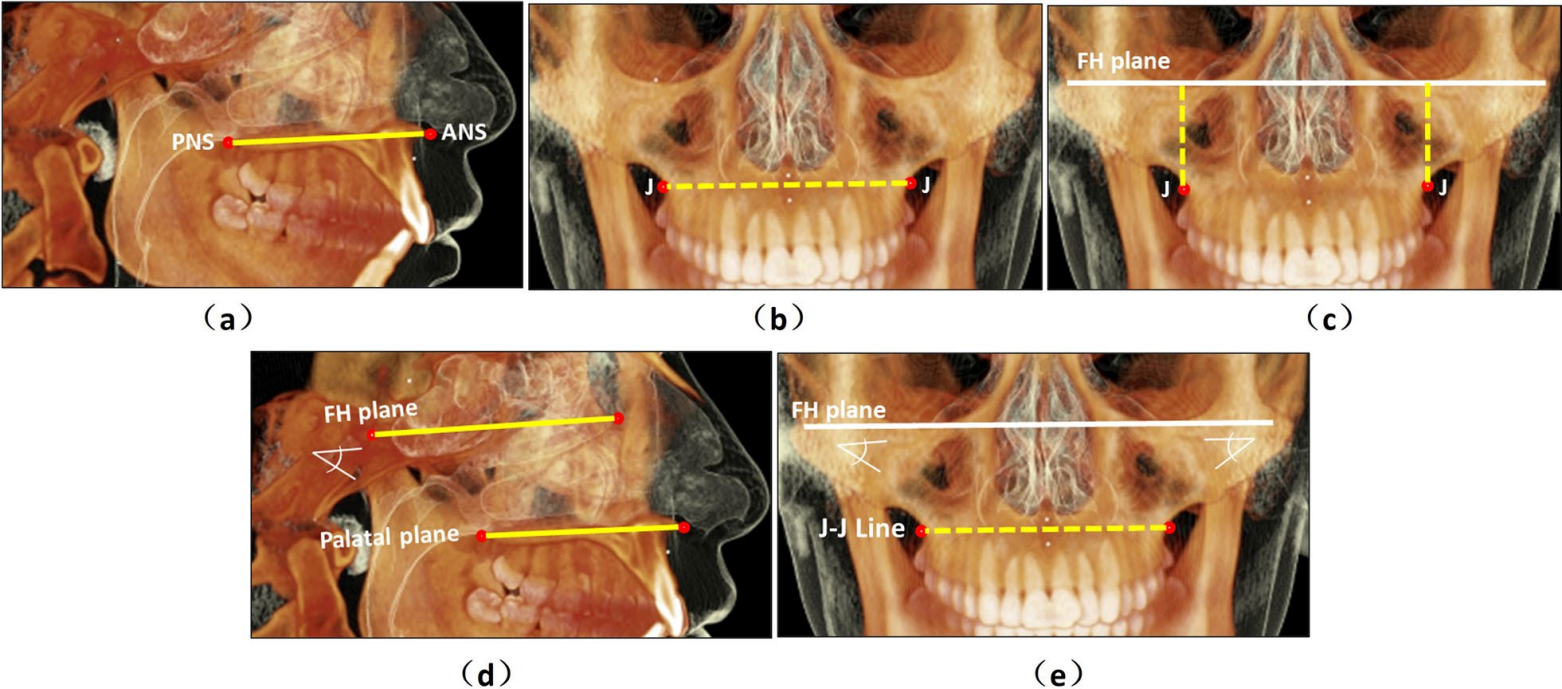
The three-dimensional cephalometric measurements of the maxilla; **a** maxillary length, **b** maxillary width, **c** maxillary height, **d** maxillary antero-posterior inclination, **e** maxillary medio-lateral inclination

The 3D analysis involved identification of anatomical landmarks (Additional file 1: Table S1), reference planes (Additional file 1: Table S2), and 3D linear and angular measurements presented in Table 2 and graphically presented in Fig. 3 [27, 41, 42].

**Table 2:** Definitions of the three-dimensional skeletal measurements of maxilla

Measurement	Abbreviation	Definition
Maxillary length	ANS-PNS (mm)	Distance between the projection of PNS and ANS points onto the sagittal plane
Maxillary width	J-J (mm)	Distance between the right and the left Jugale points (J) along the transverse axis
Maxillary height	J-FHP (mm)	The perpendicular distance from Jugale points (J) to the FH plane
Maxillary antero-posterior inclination	(PP/FHP) °	The angle between of palatal plane (PP) and Frankfort plane (FHP) antero-posteriorly onto the sagittal plane
Maxillary medio-lateral inclination	(J-J /FHP) °	The angle between J-J line and Frankfort plane (FHP) medio-lateraly onto coronal plane

With the nasion point centered as the origin of the 3D mold at the center of the three planes X, Y, and Z (coronal, axial, sagittal), then was calculated by the 3D equation of distance formula to provide a more reliable and accurate measuring:

$$d = \sqrt {\left( {{\text{x1 }}{-}{\text{ x2}}} \right){2 } + { }\left( {{\text{y1 }}{-}{\text{ y2}}} \right){2 } + { }\left( {{\text{z1 }}{-}{\text{ z2}}} \right){2}}$$

where *d* is the distance (in millimeters) between two anatomic landmarks, and × 1, y1, and z1 and × 2, y2, and z2 are the coordinates of the two landmarks at the two ends of the linear measurement.

Intra- and inter-observer reliability of three-dimensional measurements was assessed by re-measuring 10% of the sample (50 CBCTs) by two observers (W.A. and R.A.) at one-month intervals.

### Statistical analysis

IBM SPSS Statistics for Windows, Version 26.0 (Armonk, NY: IBM Corp.), was used. Intra- and inter-observer reliability analysis for the SOS scoring was calculated using Cohen's Kappa coefficient [26]. In contrast, the three-dimensional measurements' reliability was calculated by absolute and relative technical measurement errors (TEM and RTEM) and Intra-class Correlation Coefficient (ICC) test. Descriptive statistics, including each variable's mean and standard deviation, were calculated and presented. Dahlberg's formula was also used to calculate the Standard Deviation of Measurement Error (SE) [43]. Quantitative data for the normal state was explored by the verification distribution of data. Depending on Shapiro–Wilk test and Kolmogorov–Smirnov test, all groups showed a normal distribution. The data were presented as mean and standard deviation (SD) for comparative analysis.

One-way ANOVA test was used to compare between the SOS maturation stages (four SOS groups per gender: independent variables) regarding the linear and angular measures of the maxilla (dependent variables) for males and females separately. The problem of comparisons was treated by using Bonferroni correction, adjusting the *P*-value for multiple comparison tests, to avoid type I error.

The growth curves for maxillary parameters based on SOS maturation stages and chronological age were determined following a previous study [44]. *R*-statistical programing language was used for graphing and computing *R*^2^. *P*-value < 0.05 was considered statistically significant.

## Results

CBCT scans of 500 patients aged 6 to 25 years; with a mean age of 13.89 ± 1.13 years were analyzed. It included 274 females and 226 males with mean ages of 13.68 ± 5.30 and 14.14 ± 4.99 years, respectively. The distribution of subjects according to the spheno-occipital synchondrosis fusion stages, age and gender is presented in (Additional file 1: Table S3). The results of the intra- and inter-examiner reliability analysis for the SOS scoring were "almost perfect"; weighted Kappa agreement measures were more than 0.900 for each observer (Additional file 1: Table S4). Three-dimensional maxillary cephalometric measurement's reliability was "excellent agreement"; R* values of TEM and RTEM were higher than 0.95% and ICC above 98% with *P* < 0.05 (Additional file 1: Table S5).

The results showed that there was a statistically significant differences in the millimetric maxillary measurements (ANS-PNS, J-J, and J-FHP), but there was no statistically significant differences in the angular maxillary measurements (PP/FHP, and J-J/FHP) for both genders as presented in Table 3. The pair-wise comparison (Bonferroni Post hoc analysis) showed differences across different SOS stages.

**Table 3:** Descriptive statistics and the results of the One-way ANOVA test between SOS fusion stages and 3D measurements of maxillary growth pattern for males and females separately

Measurement	Gender	Stage 1	Stage 2	Stage 3	Stage 4	SOS groups Comparison One-way ANOVA
		Mean ± SD	Mean ± SD	Mean ± SD	Mean ± SD	*p*-value
ANS-PNS (mm)	M	44.15 ± 3.22^a^	46.01 ± 3.31^b^	48.85 ± 3.18^ cd^	50.34 ± 2.88^d^	0.000**
	F	42.36 ± 2.82^a^	43.47 ± 2.64^a^	45.56 ± 2.91^b^	46.98 ± 2.56^c^	0.000**
J-J(mm)	M	62.98 ± 2.94^a^	66.44 ± 3.02^ab^	67.93 ± 3.44^bc^	68.76 ± 3.53^c^	0.000**
	F	59.80 ± 3.31^a^	62.99 ± 2.93^b^	64.56 ± 2.67^ cd^	65.18 ± 3.06^d^	0.000**
J-FHP (mm)	M	25.16 ± 3.05^a^	27.01 ± 3.43^b^	29.62 ± 3.05^ cd^	30.12 ± 2.76^d^	0.000**
	F	23.32 ± 2.46^a^	25.06 ± 2.43^b^	27.73 ± 3.12^ cd^	28.13 ± 2.83^d^	0.000**
(PP/FHP) °	M	1.73 ± 1.86	1.70 ± 2.00	2.01 ± 2.17	2.25 ± 2.34	0.432
	F	2.32 ± 2.18	1.86 ± 1.85	2.16 ± 2.02	1.92 ± 1.85	0.559
(J-J/FHP) °	M	1.17 ± 0.83	1.17 ± 1.05	1.12 ± 0.80	1.35 ± 1.03	0.568
	F	1.04 ± 0.70	1.17 ± 1.00	1.29 ± 1.08	1.15 ± 0.78	0.515

^*^ Indicate significance at the 0.05 level (2-tailed). **. Indicate significance at the 0.01 level (2-tailed). (a, b, c, d) Superscripts in the same row represent a statistically significant difference between SOS stages according to multiple comparisons of Bonferroni Post hoc analysis

**Fig. 4:**
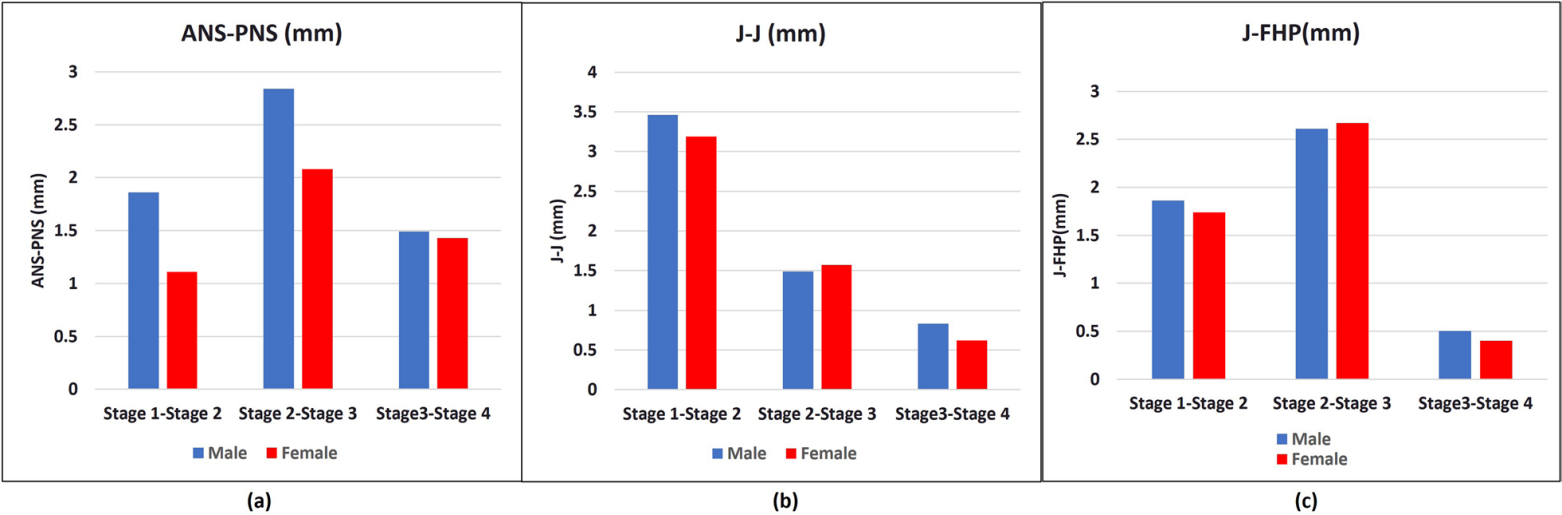
Female and male maxillary basic growth increments between the SOS maturation stages; **a** maxillary length increments, **b** maxillary width increments, **c** maxillary height increments

Regarding the mean differences in the maxillary growth among the SOS stages (Table 4 and Fig. 4); the results showed there were statistically significant mean differences between SOS stages 2 and 3, that were larger than those stages 1 and 2 and stages 3 and 4 in males and females for maxillary measurements in millimetric (ANS-PNS and J-FHP). However, the maxillary millimetric measurement) J-J (was higher between SOS stages 1 and 2 than those between stages 2 and 3, and stages 3 and 4. Otherwise, the maxillary millimetric measurement (J-J, ANS-PNS (in males), and J-FHP) had no significant mean difference between SOS stages 3–4 in both genders.

**Table 4:** Female and male maxillary growth mean differences across the SOS maturation stages

	Gender	Stage 1-Stage 2			Stage 2-Stage 3			Stage3-Stage 4			*P*- value
Measurement	Mean diff	CI 95%; Min	CI 95%; Max	Mean	diff	CI 95%; Min	CI 95%; Max	Mean	CI 95%; Min	CI 95%; Max	
ANS-PNS (mm)	M	1.86^AB^	0.25	3.47	2.84^BC^	1.23	4.45	1.49^DD^	− 0.07	3.06	*P* < 0.05*
	F	1.11^AA^	− 0.31	2.54	2.08^AB^	0.76	3.40	1.43^BC^	0.30	2.56	
J-J(mm)	M	3.46^AA^	1.79	5.13	1.49^AB^	− 0.18	3.16	0.83^CC^	− 0.80	2.45	
	F	3.19^AB^	1.61	4.76	1.57^BC^	0.12	3.03	0.62^DD^	− 0.62	1.87	
J-FHP (mm)	M	1.86^AB^	0.28	3.43	2.61^BC^	1.03	4.18	0.50^DD^	− 1.03	2.04	
	F	1.74^AB^	0.28	3.20	2.67^BC^	1.32	4.02	0.40^DD^	− 0.76	1.55	

Bold value indicates *P* < 0.05

^* ^Indicate significance at the 0.05 level (2-tailed). (A, B, C, D) Superscripts in the same row represent a statistically significant difference between SOS stages according to multiple comparisons of Bonferroni Post hoc analysis

**Fig. 5:**
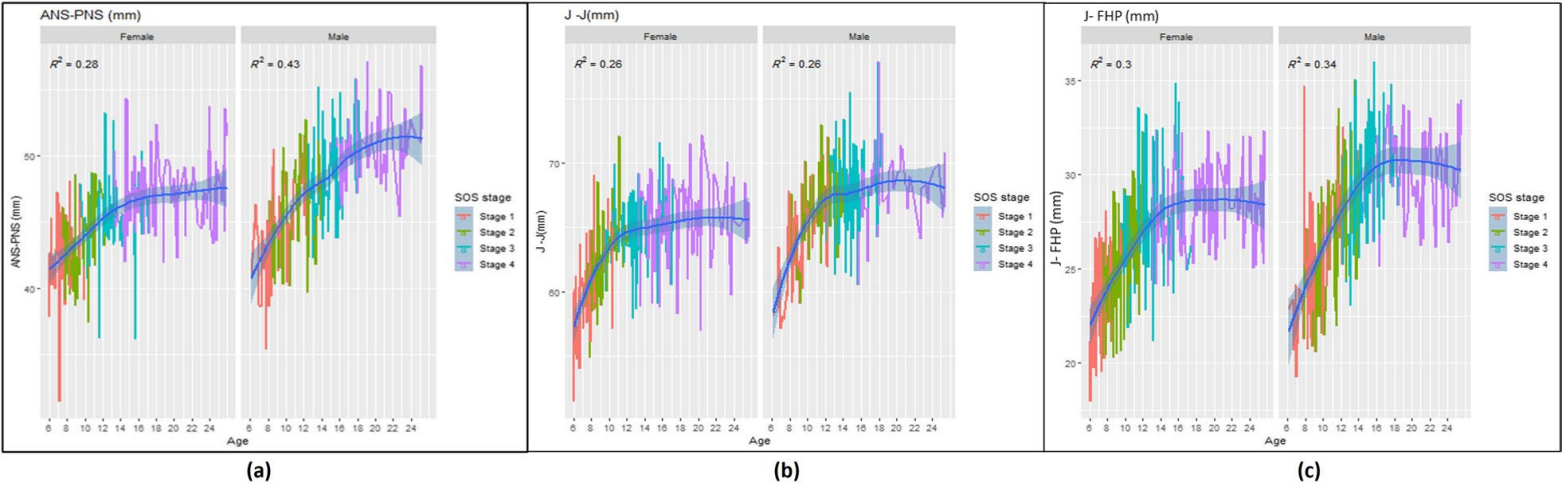
Female and male maxillary basic growth curves according to the SOS maturation (stages1-4) and chronological age (6 through 25 years); **a** maxillary length curve, **b** maxillary width curve, **c** maxillary height curve

The basic maxillary growth curves according to SOS stages, chronological age for females and males with the effect size *R*^2^ and *P* < 0.05 are graphically presented in Fig. 5**.**

## Discussion

The evaluation of skeletal maturation of craniofacial complex has critical importance in orthodontic, and dentofacial orthopedic. Recently, the SOS method has been considered as a reliable tool and correlated well with other established methods; Hand-wrist maturation and CVM index in assessment of skeletal age [10, 18, 22, 23, 25, 45–49]. However, no study in the available literature related to the three-dimensional maxillary growth and the SOS fusion stages.

The superiority of SOS method may relate to the critical location of SOS in cranial base. In which its late ossification and contribution to post-natal cranial base growth play critical role in facial development [1, 2, 50–52]. Moreover, the CBCT have been used widely in dental field, so the SOS may be considered as suitable method as CBCT provides the benefits of low-cost, high-resolution, accurate three-dimensional imaging (3D) without the risk of increased radiation exposure to the patient, and easy visualization of superimposed bony structures [53]. On the other hand, Hand -wrist method requires expert knowledge and expenditure of time by the operator, and their accuracy is not very high. It also had the drawback of unnecessary radiographic dose in an area away from the area of interest [11].

There were significant differences in the mean maxillary linear parameters among SOS maturation stages for males and females as the following: maxillary length, width, and height. However, there was no statistically significant difference for maxillary angular parameters in anteroposterior and mediolateral directions. This might indicate that the SOS maturation stages have similar proportional growth increases with significant maxillary parameters. So, this may support the use of the SOS maturation stages as a valid method to assess the three-dimensional maxillary growth. This finding is comparable with gold standard method of validity of CVM method for assessment of facial growth [18, 48].

The mean differences in the maxillary growth between SOS stage 2 and 3 were larger than those between stages 1and 2 or stages 3 and 4 in males and females for maxillary length and height. This might indicate that the maxillary growth peak is between stages 2 and 3. This finding may be supported by the theory that spheno-occipital synchondrosis begins to fuse around growth puberty [22, 24, 35]. However, the maxillary width’s mean difference was higher between SOS stages 1 and 2 than those stages 2 and 3, and stages 3 and 4. This finding may reinforced by what reported in literature about facial growth sequence [54]; the sequential completion of the cranium followed by facial width, then facial depth and height. Otherwise, the maxillary millimetric measurement (width, length (in males), and height) had no significant mean difference between SOS stages 3–4 in both genders. This may indicate a lesser growth during this period and agree with theory of the earlier maturation of maxilla for mentioned parameters [55, 56].

The basic growth curves of maxillary parameters were constructed based on SOS maturation stages and chronological age in both genders as the following: the maxillary length, width, and height had increased with increasing chronological age, early in females than males. But the increasing for these parameters with SOS’s fusing were in stages 1, 2, and 3 (primarily accelerated in stages 2 and 3); they then tended to be steadier in stage 4 for males and females in a similar pattern. These findings may indicate that the maxillary parameters had maximum growth as the SOS was still fusing than the fused stage (stage 4) for males and females. That is comparable with Jabour and Anwar Shawqi's finding of mandibular length and SOS fusing stages [22].

Moreover, there was sexual dimorphism in mentioned maxillary growth curves as the fusion of SOS and growth of maxilla were earlier chronological age in females than males. This sexual dimorphism consistent with previous research that reported the SOS fuses earlier in females than in males [24, 26, 31, 32]. The effect size (R^2^) of SOS fusion stages on the maxillary length, width, and height ranged from 19 to 36% for females and from 32 to 39% for males. These percentages represent the variations of these maxillary parameters due to the SOS maturation stages. According to previous studies [13, 57], the *R*^2^ considered high for biological data (based on the CVM method) when it ranges from 30 to 67%. So, this may reflect the applicability of the presented growth curves as the effect size of SOS on the maxillary parameters is relatively moderate to high for females and high for males.

As a clinician, it is worthy to mention that regarding the clinical implications of using the SOS fusion stages as a maxillary skeletal indicator; this study was designed to compare different categories of ages based on the expected maxillary growth changes during these different ages and because age is a weak determinant, a more standardized and well-established method was selected to answer this question (SOS) so that the clinician can decide whether to proceed with the growth modification mechanics or to wait for proper stage of intervention.

In this study, the overall view about the three-dimensional maxillary growth during SOS fusion stages in both genders, assessing the reliability of the SOS method as a skeletal indicator of 3D maxillary growth, and 3D maxillary growth spurt based on SOS fusion stages, which aren’t available in the published literature is now presented. This may have an importance in orthodontic diagnosis or considered as a base for further research in the future.

The clinical application of these findings suggests that if SOS is still fusing, the individual would have the maximum maxillary growth in width, length, and height. Moreover, the maxillary growth completion based on SOS fusion stages and chorological age follow sequences of width, then length and height**.** So, these findings may help understand the three-dimensional growth pattern of the maxilla according to the SOS maturation stages during treatment planning for orthodontic, dentofacial orthopedic, or orthognathic treatment planning.

The limitation of this study starts with its nature as a cross-sectional study; there is no doubt that the longitudinal studies of the maxillary growth and development provide a more thorough understanding. However, the challenges of acquiring high sample numbers for a longitudinal study, the related increase in the number of radiographic exposures during the follow up time, and the ethical considerations are likely to rule out this approach and to use the cross-sectional direction as an alternative. Also, this study might be considered as primary reporting in this field, and we hope there will be more detailed studies in future of a longitudinal design. Another limitation is that the ethnic group is limited to the Chinese population, making it less practical for other ethnicities. The sample had no skeletal classes or facial pattern specifications, which might affect the current findings. Despite the advantages of CBCT of low-cost, high-resolution, accurate three-dimensional imaging (3D) without the risk of increased radiation exposure to the patient, easy visualization of superimposed bony structures, and the highly used in the dental field, detailed description of the SOS method can only be done with CBCT imaging, and it is not very obvious in plain x- ray, but the use of this technology should be based on the benefit risk ratio. When this is the case, other problems should be there for cases refereed to this imaging modality or specific CBCT parameters are to be set for this purpose.

## Conclusions

The SOS maturation stages are valid and reliable maxillary skeletal maturation indicators for three-dimensional maxillary growth in both genders.

The mean differences in the maxillary growth between SOS stages 2 and 3 were higher than those between stages 1and 2 and stages 3and 4 for maxillary length and height in both genders. However, the maxillary width’s mean difference was higher between SOS stages 1 and 2 than those stages 2 and 3 and stages 3 and 4. On other hand, there may be lesser maxillary growth between SOS stages 3 and 4 for maxillary width, length (in males), and height.

The basic growth curves showed high active growth of the maxilla as the SOS was still fusing (especially stage 2 and 3) than those of the fused (stage 4). Moreover, the acceleration of growth occurred earlier in females than males regarding chronological age but not for SOS maturation stages.

## Supplementary Information

**Additional file 1: Table S1.** Definitions of the maxillary three-dimensional skeletal landmarks; **Table S2.** Definitions of the three-dimensional craniofacial reference planes and lines; **Table S3.** Distribution of subjects according to spheno-occipital synchondrosis fusion stage, age and gender; **Table S4.** The reliability SOS staging; **Table S5.** Reliability analysis of three-dimensional maxillary measurements.

## Data Availability

All data and materials are available on the Orthodontics Department of stomatology school, Lanzhou University, China. Please get in touch with the corresponding authors for any requests.

## Abbreviations

SOS: Spheno-occipital synchondrosis
DICOM: Digital imaging and communications in medicine
CBCT: Cone beam computed tomography
CT: Computed tomography
3D: Three-dimensional
ICC: Intra-class correlation coefficient
