# Supplementary material for: Comparison of three-dimensional maxillary growth across spheno-occipital synchondrosis maturation stages
Source: BMC Oral Health. 2023 Feb 14;23:100. doi: 10.1186/s12903-023-02774-w (PMC9930258; doi:10.1186/s12903-023-02774-w)
Supplement: Supplementary file 1 — Additional file 1: Table S1. Definitions of the maxillary three-dimensional skeletal landmarks; Table S2. Definitions of the three-dimensional craniofacial reference planes and lines; Table S3. Distribution of subjects according to spheno-occipital synchondrosis fusion stage, age and gender; Table S4. The reliability SOS staging; Table S5. Reliability analysis of three-dimensional maxillary measurements. [file 12903_2023_2774_MOESM1_ESM.docx]

**Supplementary table 1.** Definitions of the maxillary three-dimensional skeletal landmarks.

| **Landmark** | **Definition** |
| --- | --- |
| **Orientation landmarks:** | |
| Basion (Ba) | The most anterior and inferior point of the foramen magnum. |
| Nasion (N) | The midpoint of the frontonasal sutures. |
| Porion (Po) | The most superior point of external auditory meatus. |
| Orbitale (Or) | The most inferior point of infra-orbital rim |
| **Maxillary landmarks:** | |
| Anterior Nasal Spine (ANS) | The most anterior midpoint of the maxillary anterior nasal spine. |
| Posterior Nasal Spine (PNS) | The most posterior midpoint of the palatine bone in the posterior nasal spine. |
| A point (A) | The point of maximum concavity in the mid-line of the alveolar process of the maxilla. |
| Jugale point (J) | The intersection of the lateral contour of the maxillary alveolar process and the lower contour of the maxillozygomatic (jugal) process of the maxilla. |

**Supplementary table 2.** Definitions of the three-dimensional craniofacial reference planes and lines.

| Plane and lines | Definition |
| --- | --- |
| Frankfurt horizontal palnw(FHP) | The horizontal plane (X) was passing through the right and left porions and the right orbitale. |
| Mid-saggitl plane (MSP) | The mid-sagittal plane (Y) was passing through nasion and basion and perpendicular to the FH plane. |
| Coronal plane(CP) | The coronal plane (Z) was passing through sella turcica and perpendicular to the midsagittal plane and FH plane. |
| Palatal plane( PP) | The plane that passes ANS and PNS landmarks and is perpendicular to the mid-sagittal plane. |
| J-J Line | The line that connect between right and left juglae points. |

**Supplementary table 3.** **Distribution of subjects according to spheno-occipital synchondrosis fusion stage, age and gender**

| **Stages** | **N** | **Mean age** | **SD** | **95% CI** | **Median age** | **Min-Max** |
| --- | --- | --- | --- | --- | --- | --- |
| Females | 274 |  |  |  |  |  |
| Stage 1 | 48 | 7.44 | 1.18 | 7.10-7.79 | 7.30 | 6.00-11.60 |
| Stage2 | 54 | 9.62 | 1.25 | 9.28-9.96 | 9.50 | 7.60-12.20 |
| Stage 3 | 66 | 12.94 | 1.75 | 12.51-13.37 | 12.75 | 9.50-16.90 |
| Stage 4 | 106 | 19.03 | 3.78 | 18.30-19.76 | 18.75 | 13.20-25.90 |
| Males | 226 |  |  |  |  |  |
| Stage 1 | 49 | 8.79 | 1.49 | 8.36-9.22 | 8.50 | 6.10-12.30 |
| Stage 2 | 60 | 11.13 | 1.64 | 10.71-11.55 | 11.50 | 7.80-13.90 |
| Stage 3 | 49 | 14.82 | 1.72 | 14.32-15.31 | 14.80 | 11.70-18.30 |
| Stage 4 | 68 | 20.18 | 3.33 | 19.37-20.98 | 20.20 | 14.00-25.50 |
| Total | 500 |  |  |  |  |  |
| CI: confidence interval; SD: standard deviation | | | | | | |

**Supplementary table 4.** The reliability SOS staging.

|  | Intra-observers | | Interobservers |
| --- | --- | --- | --- |
|  | W.A. | R.A. | Both |
| Number of cases | 100 | 100 | 500 |
| Weighted Kappa (k) | 0.922 | .922 | 0.900 |
| Significance | P < 0.001 | P < 0.001 | P < 0.001 |

**Supplementary table 5.** Reliability analysis of three-dimensional maxillary measurements.

| **Measurements** | **Inter-observer reliability** | | | | **Intra-observer reliability** | | | |
| --- | --- | --- | --- | --- | --- | --- | --- | --- |
|  | **ICC** | **TEM** | **RTEM** | **R** | **ICC** | **TEM** | **RTEM** | **R** |
| ANS-PNS (mm) | 0.9983 | 0.2722 | 0.2569 | 0.9893 | 0.9985 | 0.2204 | 0.2085 | 0.9931 |
| J -J(mm) | 0.9979 | 0.2881 | 0.1940 | 0.9898 | 0.9984 | 0.2413 | 0.1626 | 0.9927 |
| (PP/FHP) ° | 0.9904 | 0.3452 | 8.8854 | 0.9575 | 0.9970 | 0.1984 | 5.2237 | 0.9858 |
| (J-J/FHP) ° | 0.9877 | 0.2247 | 7.5568 | 0.9582 | 0.9992 | 0.0674 | 2.2677 | 0.9963 |
| J-FHP (mm) | 0.9972 | 0.3524 | 0.5618 | 0.9853 | 0.9986 | 0.2403 | 0.3816 | 0.9931 |

Note: TEM and rTEM indicate an absolute and relative technical error of measurement. ICC indicates the interclass correlation. All R* values were higher than the 0.95 percent indicated cut-off.
